# Health Resorts

**Published:** 1874-04

**Authors:** 


### HEALTH RESORTS.

### HOT SPRINGS OF ARKANSAS.

THESE springs are fifty-five miles southwest of Little Rock, the capital of the State, and twenty-one miles from Malvern (the nearest railroad station), on the Cairo and Fulton R. R.

There are fifty-seven springs, which issue from the western slope of the Hot Spring Mountain, at an elevation of 1,300 to 1,400 feet above the level of the sea. Most of them are 50 to 100 feet above the level of the valley. These waters flow into a beautiful mountain stream, twenty to thirty feet wide. All the springs on the east side of the creek (with one exception) are hot, and all on the west side (except the Alum Spring) are cold.

The water of the springs is conveyed through pipes to the bathing houses in the valley.

The waters contain the following ingredients: Alumina and Oxide of Iron, Bicarb. of Lime, Bicarb. of Magnesia, Carb. of Soda, Carb. of Potash, Chloride of Potassium, Chloride of Sodium, Chloride of Magnesia, Sulph. of Lime, Sulph. of Magnesia. Their medicinal virtue is mainly due to their high temperature and abundant supply. After being conducted in open troughs down the hillside they are as hot as can be borne; and the waste water, conducted under the vapor bath houses, sends up a steam through the latticed floor of a temperature so hot that few can endure it.

If the Warm Springs of Virginia, which have a temperature of only 96 deg. to 98 deg., exercise, as experience has proved, a most potent effect in the cure of many diseases, "mainly by their temperature," how much more positive must be the effect of waters of so much higher temperature; especially when a stream of it, in diameter as large as a man's arm, can be directed at pleasure, with great force, on any organ.

In many forms of chronic diseases especially, its effects are truly astonishing. The copious diaphoresis which the hot bath establishes, opens in itself a main channel for the expulsion of principles injurious to health. A similar effect, in a diminished degree, is also effected by drinking the hot water—a common, indeed almost universal practice, among invalids at the Hot Springs.

The impression produced by the hot douche, as above described, is indeed powerful, arousing into action sluggish and torpid secretions. The languid circulation is thus purified of morbific matters, and thereby renewed vigor and healthful action are given both to the absorbents, lymphatics, and to the excretory apparatus—a combined effect which no medicine is capable of accomplishing.

Silica and carbonate of lime, the most abundant mineral constituents of the Hot Springs, can have comparatively little specific action on the animal functions. The carbonates of alkalies present, proved by the distinct alkaline reaction of the watery solution of the solid contents evaporated to dryness, cannot be without their therapeutic effects, in common, however, with a great many of the well and spring waters of middle and southern Arkansas, which also contain some alkaline carbonates.

The large quantity of free carbonic acid which the water contains, and which rises in volumes through the water at the fountain of many of the springs, has, undoubtedly, an exhilarating effect on the system; and it is, no doubt, from the water of the Hot Springs coming to the surface charged with this gas, that invalids are enabled to drink it freely at a temperature at which ordinary tepid water, from which all the gas has been expelled by ebullition, would act as an emetic.

The small quantities of chlorides and sulphates of magnesia may have a slight medicinal effect; but there are not more of these salts present than are to be found in many spring and well waters employed for domestic purposes.

These waters resemble the waters of Gastien, in Austria, and Pfeffers, in Switzerland. They are very highly esteemed, and deservedly so, in the treatment of Chronic Rheumatism, Gout, Contractions of Joints, Secondary and Tertiary Syphilis and Neuralgia. In Paralysis, unaccompanied by organic lesions, they are of considerable utility as auxiliaries. In dartrous diseases of the skin, functional diseases of the uterus, and chronic poisoning by metals, either lead or mercury, they are efficient. Experience proves them to be *positively injurious* in affections of the heart or brain, or dropsies of the lungs in any form, and persons laboring under diseases for which these waters are beneficial, but accompanied by such maladies, need not journey to the Hot Springs.

How do these waters act? Having a continuous flow of three hundred and sixty gallons per minute, and ranging in temperature from 93 deg. to 150 deg. Fahrenheit, we would expect favorable results from their judicious use; and we are not surprised to learn of cures under their employment that have resisted all other modes of treatment. It is asked, "Why not use hot water at home?" Because it is impossible to procure it in sufficient quantity and of uniform temperature. Some consider that terrestrial heat possesses peculiar properties, rendering it more efficient than artificial.

### CUTANEOUS DISEASES.

Diseases of the skin, especially those of a specific type, Chronic Rheumatism, Gout, Rheumatic Gout, Scrofula, Syphilitic and Mercurial Diseases, are those that derive the greatest benefit from a proper use of the baths.

Speedy cures have been made of many long standing cases of suppurating disease of the glands of the neck; large non-suppurating swellings of these glands, that had endured for years, have been reduced to a healthy size and condition; Chronic Ague, and its attendant enlargement of the spleen; Alcoholism, or the morbid effects traceable to the abuse of stimulants containing alcohol; Migraine, or Sick Headache; Paralysis, especially when of specific or venereal origin; Acute and Chronic Chorea, Hysteria, Neuralgic Affections; Ozena, Chronic Nasal Catarrh, Sore Throat, Ulcerated Throat, Relaxed Throat, Scrofulous Disease of Tonsils, Enlarged Tonsils, Painful Menstruation, Scanty Menstruation, Absent Menstruation, from want of development at time of puberty and from temporary suppression; Ulcer of the Womb, Chlorosis, or Green Sickness; Rheumatism of the Uterus, Chronic Inflammation of the Bladder.

### CAUTION.

Patients suffering from Phthisis, Dropsy, Acute Inflammatory Affections, Aneurisms, and other diseases of the heart, brain and large blood vessels, cannot expect relief.

Winter bathing, in the majority of cases, is just as efficacious as that in summer; and the mild climate of this latitude ($34\frac{1}{2}$ deg.) makes it, especially to northern visitors, a most desirable winter resort.

SUGGESTIONS TO INVALIDS.

Persons afflicted with any of the diseases likely to be relieved or cured by the hot baths, should consult with some competent physician at home, to ascertain whether there is not some other disease in the system which the baths might hasten to a fatal termination. By observing this precaution the invalid may be saved the fatigue and expense of a long, weary journey, and the sore disappointment of ascertaining, on his arrival at the Springs, what he should have learned before starting, that the baths, instead of benefiting, would probably prove fatal to him.

HOTELS.

There are sixteen hotels and as many boarding houses at Hot Springs. The price of board and rooms ranges from $35 to $75 per month. One hotel charges $60 per month (baths included).

For information contained in this article we are indebted to Mr. Charles Cutter, author of a pamphlet entitled "The Hot Springs as They Are."

